# Research Issues: A Question of Balance

**Published:** 2006-11

**Authors:** Charles W. Schmidt

Expert committees convened by the National Academy of Sciences (NAS) have long advised the U.S. government and the public on challenging technical issues. But are those committees fair and balanced in their views? Perhaps not, according to the Center for Science in the Public Interest (CSPI), a Washington, DC–based advocacy group. In an investigation spanning two and a half years, the CSPI reviewed 21 NAS committees and concluded that one in five of their members had direct ties to industries with a stake in study outcomes. The group reports its findings in *Ensuring Independence and Objectivity at the National Academies*, released 24 July 2006.

Merrill Goozner, director of CSPI’s Integrity in Science Project and a coauthor of the report, acknowledges the investigation could find no evidence showing that NAS conclusions were adulterated by industry affiliations. “Nevertheless,” he says, “the report raises an important question: do industry ties make committees less bold with respect to their conclusions than they might be otherwise?”

CSPI’s conclusions hinge on its controversial definition of “conflict of interest,” described in the report as “a financial tie within the last five years to a company or industry that is relevant to a committee topic.” Of 320 experts reviewed by CSPI, at least 56 had conflicts meeting that criterion, while 66 had a history of espousing what the authors call “pro-industry” positions in research papers or legal testimony. Nine experts were closely aligned with nonprofit environmental or public-interest organizations.

The NAS responds that, if applied, CSPI’s conflict-of-interest definition would make it nearly impossible to recruit qualified experts for committee membership. Spokesman Bill Kearney says the NAS merely considers whether experts or their immediate relations or business partners have *current* financial interests that might be directly affected by the committee’s work. Previous ties to industry—or to any other interest—don’t influence the selection, he says, unless they reveal a distinct bias towards a particular view, which can be balanced by adding someone with an opposing view to the committee.

“We disagree with CSPI’s definition,” Kearney says. “It excludes people who in our opinion don’t have a financial stake in the outcome of a study.” Kearney also stresses that the NAS’s conflict-of-interest screens comply with the Federal Advisory Committee Act.

Dan Greenbaum, president of the private Health Effects Institute, serves on the NAS Board on Environmental Studies and Toxicology, and has also participated in expert committees on air quality. He warns that CSPI’s more stringent criteria could exclude some outstanding and impartial scientists, and that fuller public disclosure could raise issues of privacy. But he adds that the report serves a useful purpose by pushing for conflict-of-interest data at earlier stages in the selection process. “CSPI is helping to move that agenda forward,” he says. “They’ve made the academy nervous and at times understandably upset . . . but they’ve also brought in a level of scrutiny that advances the process.”

